# Access to and Affordability of World Health Organization Essential Medicines for Cancer in Sub-Saharan Africa: Examples from Kenya, Rwanda, and Uganda

**DOI:** 10.1093/oncolo/oyac143

**Published:** 2022-08-22

**Authors:** Darya A Kizub, Sachin Naik, Ayokunle A Abogan, Debanjan Pain, Stephen Sammut, Lawrence N Shulman, Yehoda M Martei

**Affiliations:** Department of General Oncology, Division of Cancer Medicine, University of Texas MD Anderson Cancer Center, Houston, TX, USA; Harvard Medical School, Boston, MA, USA; Clinton Health Access Initiative, Boston, MA, USA; Department of Medicine (Hematology-Oncology), University of Pennsylvania, Philadelphia, PA, USA; Department of Health Care Management, Wharton School, University of Pennsylvania, Philadelphia, PA, USA; Department of Medicine (Hematology-Oncology), University of Pennsylvania, Philadelphia, PA, USA; Department of Medicine (Hematology-Oncology), University of Pennsylvania, Philadelphia, PA, USA

**Keywords:** antineoplastic agents, healthcare financing, costs and cost analysis, Kenya, Uganda, Rwanda

## Abstract

**Background:**

Cancer mortality is high in sub-Saharan Africa (SSA), partly due to inadequate treatment access. We explored access to and affordability of cancer treatment regimens for the top 10 cancers utilizing examples from Kenya, Uganda, and Rwanda.

**Materials and Methods:**

Population, healthcare financing, minimum wage, and cancer incidence and mortality data were obtained from the WHO, World Bank, public sources, and GLOBOCAN. National Essential Medicines List (NEML) alignment with 2019 WHO EML was assessed as a proportion. Cancer regimen pricing was calculated using public and proprietary sources and methods from prior studies. Affordability through universal healthcare coverage (UHC) was assessed as 1-year cost <3× gross national income per capita; and to patients out-of-pocket (OOP), as 30-day treatment course cost <1 day of minimum wage work.

**Results:**

A total of 93.4% of the WHO EML cancer medicines were listed on the 2019 Kenya NEML, and 70.5% and 41.1% on Uganda (2016) and Rwanda (2015) NEMLs, respectively. Generic chemotherapies were available and affordable to governments through UHC to treat non-Hodgkin’s lymphoma, cervical, breast, prostate, colorectal, ovarian cancers, and select leukemias. Newer targeted agents were not affordable through government UHC purchasing, while some capecitabine-based regimens were not affordable in Uganda and Rwanda. All therapies were not affordable OOP.

**Conclusion:**

All cancer treatment regimens were not affordable OOP and some were not covered by governments. Newer targeted drugs were not affordable to all 3 governments. UHC of cancer drugs and improving targeted therapy affordability to LMIC governments in SSA are key to improving treatment access and health outcomes.

## Teaser

This study evaluated the access to, and affordability of treatment regimens based on 2019 WHO Essential Medicines List (EML) indications for the 10 most common cancers in Kenya, Uganda, and Rwanda. The findings demonstrate that all cancer treatment regimens were unaffordable to patients paying out-of-pocket and that novel essential targeted agents were not always available on country EMLs and were unaffordable to governments in all 3 countries through universal healthcare coverage purchasing.

**Implications for Practice**

Cancer mortality is high in Sub-Saharan Africa (SSA), in part due to inadequate treatment access. We evaluated the access to and affordability of treatment regimens based on 2019 WHO Essential Medicines List (EML) indications for the 10 most common cancers in Kenya, Uganda, and Rwanda using country-specific healthcare metrics. All cancer treatment regimens were unaffordable to patients paying out-of-pocket. Novel essential targeted agents were not always available on country EMLs and were unaffordable to governments in all 3 countries through universal healthcare coverage purchasing. These findings highlight the importance of universal healthcare coverage and ensuring the affordability of both novel and standard-of-care essential cancer medicines to both governments and patients in improving cancer outcomes in SSA and in other low and middle-income countries.

## Introduction

In 2020, an estimated 11 of 19 million new cancer cases (59.1%) and 7 of 10 million (71.0%) cancer deaths, occurred in low- and middle-income countries (LMICs).^1^ Sub-Saharan Africa (SSA) has a disproportionate burden of cancer mortality with the annual number of cases projected to increase from 727 000 to 1.4 million from 2020 to 2040, while annual mortality will more than double from 484 000 to 967 000 during this same time period.^1^ Reasons for the high case fatality rate in SSA are multifactorial and partly due to the patient and diagnostic delays leading to advanced cancer stage at diagnosis and suboptimal access to treatment.^2-10^

The World Health Organization’s (WHO) essential medicines list (EML) includes drugs deemed to be safe and effective in meeting the needs of a healthcare system. Many countries use the WHO EML to prioritize medicines for inclusion in national essential medicine lists (NEML), which is the first step to improving access to medications, including cancer drugs. However, the inclusion of a drug on the WHO EML does not guarantee its inclusion in NEMLs or availability at the point of care delivery.^11^ While the WHO EML traditionally included generic chemotherapies and hormone therapies, the more updated EMLs include newer and more expensive cancer medicines supported by clinical trial data showing the superior efficacy of these drugs despite their high costs.^12-14^ Even when a drug is included in the NEML, barriers to access include high out-of-pocket costs, drug stockouts, and inefficiencies in procurement and other chemotherapy supply chain challenges.^15^

The 2019 WHO EML included 61 recommended antineoplastic and supportive agents to treat 28 disease cancer sites in adults (Table 1).^12,13^ Many of the drugs that were recently added will continue to be on patent or will come off patent in the next couple of years. It is expected that the inclusion of higher-cost therapies in the WHO EML will result in strategies to negotiate the cost of these drugs.^16^

**Table 1.** 2019 List of WHO essential medicines with indications for cancer treatment and alignment with NEMLs.

Medicine	2019 WHO EML indication	Kenya EML 2019	Uganda EML 2016	Rwanda EML 2015
Arsenic trioxide*	Acute promyelocytic leukemia (APL)	+	0	0
Asparaginase*	Acute lymphoblastic leukemia (ALL)	+	+	0
Bendamustine*	Follicular lymphoma (FL), chronic lymphocytic leukemia (CLL)	+	0	0
Bleomycin	Testicular germ cell tumor, ovarian germ cell tumor, Hodgkin lymphoma, Kaposi’s sarcoma	+	+	+
Capecitabine*	Early-stage colon cancer, early-stage rectal cancer, metastatic colorectal cancer, metastatic breast cancer, ovarian germ cell tumors, osteosarcoma, retinoblastoma	+	+	0
Carboplatin	Osteosarcoma, retinoblastoma; local and metastatic breast cancer, cervical cancer, ovarian cancer, local head, and neck cancer	+	+	+
Chlorambucil	CLL	+	+	0
Cisplatin*	Epithelial ovarian cancer, early-stage cervical cancer, head and neck cancer, testicular germ cell tumor, ovarian germ cell tumor, non-small cell lung cancer, osteosarcoma	+	+	+
Cyclophosphamide	CLL, diffuse large B-cell lymphoma (DLBCL), early-stage and metastatic breast cancer, gestational trophoblastic neoplasia, Hodgkin lymphoma, FL, Burkitt’s lymphoma, rhabdomyosarcoma, Ewing sarcoma, ALL, multiple myeloma	+	+	+
Cytarabine	Acute myelogenous leukemia (AML), APL, ALL, Burkitt’s lymphoma	+	+	0
Dacarbazine	Hodgkin lymphoma	+	+	0
Dactinomycin	Gestational trophoblastic neoplasia, rhabdomyosarcoma, Ewing sarcoma, Wilms tumor	+	+	0
Daunorubicin	AML, APL, ALL	+	+	0
Docetaxel	Early-stage breast cancer, metastatic breast cancer, metastatic prostate cancer	+	+	+
Doxorubicin	Epithelial ovarian cancer, DLBCL, early-stage and metastatic breast cancer, Hodgkin lymphoma, Kaposi’s sarcoma, FL, osteosarcoma, Ewing sarcoma, acute lymphoblastic leukemia, Wilms tumor, Burkitt’s lymphoma	+	+	+
Etoposide	Epithelial ovarian cancer, testicular germ cell tumor, gestational trophoblastic neoplasia, Hodgkin lymphoma, non-small cell lung cancer, ovarian germ cell tumor, retinoblastoma, Ewing sarcoma, ALL, Burkitt’s lymphoma	+	+	0
Fludarabine*	CLL, AML	0	0	0
Fluorouracil	Early-stage breast cancer, early-stage and metastatic colon cancer, early-stage rectal cancer	+	+	+
Gemcitabine*	Epithelial ovarian cancer, non-small cell lung cancer, metastatic breast cancer	+	+	0
Hydroxycarbamide	Chronic myeloid leukemia (CML)	+	+	+
Ifosfamide	Testicular germ cell tumor, ovarian germ cell tumor, osteosarcoma, Rhabdomyosarcoma, Ewing sarcoma, AML	+	+	+
Irinotecan*	Metastatic colorectal cancer	+	+	+
Mercaptopurine	ALL, APL	+	+	+
Methotrexate	Early-stage and metastatic breast cancer, gestational trophoblastic neoplasia, osteosarcoma, ALL, APL	+	+	+
Thioguanine	ALL	+	+	0
Oxaliplatin	Local and metastatic colon cancer, metastatic rectal cancer	+	+	+
Paclitaxel	Local and metastatic breast cancer, local and metastatic epithelial ovarian cancer, ovarian germ cell tumors, Kaposi sarcoma, nasopharyngeal/head and neck cancer, non-small cell lung cancer, cervical cancer	+	+	+
Procarbazine	Hodgkin lymphoma	+	+	0
Vinblastine	Testicular germ cell tumors, ovarian germ cell tumors, Hodgkin lymphoma	+	+	0
Vincristine	Retinoblastoma, Rhabdomyosarcoma, Ewing sarcoma, ALL, Nephroblastoma (Wilms tumor), Burkitt lymphoma, Hodgkin lymphoma, DLBCL, Kaposi sarcoma	+	+	+
Vinorelbine	Non-small cell lung cancer, metastatic breast cancer	+	0	0
Allopurinol	Tumor lysis syndrome	+	+	+
Calcium folinate (Leucovorin)	Osteosarcoma, Burkitt’s lymphoma, early-stage colon cancer, early-stage rectal cancer, gestational trophoblastic neoplasia	+	+	+
Filgrastim (G-CSF)*	Primary and secondary prophylaxis	+	+	0
Mesna	Testicular germ cell tumor, ovarian germ cell tumor, osteosarcoma, soft-tissue sarcoma, rhabdomyosarcoma, Ewing sarcoma	+	+	0
Anastrozole (class)	Early-stage breast cancer, metastatic breast cancer	+	+	+
Bicalutamide	Metastatic prostate cancer	+	+	0
Dexamethasone	Ovarian germ cell tumor, ALL, Burkitt’s lymphoma, multiple myeloma; metastatic prostate cancer	+	+	+
Diethylstilbesterol	Metastatic prostate cancer	+	+	0
Hydrocortisone	ALL	+	+	+
Methylprednisolone	ALL	+	+	0
Leuprolide (class, includes goserelin)	Metastatic prostate cancer; early-stage and metastatic breast cancer	+	+	0
Tamoxifen	Local and metastatic breast cancer	+	+	+
Prednisolone	ALL, Burkitt lymphoma, Hodgkin lymphoma, DLBCL	+	+	+
Imatinib	CML, GI stromal tumor	+	0	+
Trastuzumab	Early-stage and metastatic HER2+ breast cancer	+	0	+
Rituximab	Imatinib-resistant CML	+	+	+
Cancer medicines added in 2017				
Bortezomib	Multiple myeloma	+	0	0
Zoledronic acid	Multiple myeloma	+	0	+
Nilotinib	CML	+	0	0
Dasatinib	Imatinib-resistant CML	0	0	0
Cancer medicines added in 2019				
Melphalan	Multiple myeloma	+	0	+
Lenalidomide	Multiple myeloma	+	0	0
Thalidomide	Multiple myeloma	+	+	0
Pegasparaginase	ALL	0	0	0
Realgar-Indigo naturalis	APL	0	0	0
Abiraterone/prednisone	Metastatic prostate cancer	+	0	0
Erlotinib or gefitinib	Non-small cell lung cancer	+	0	0
Nivolumab or pembrolizumab	Metastatic melanoma	+	0	0
TOTAL		57/61	43/61	25/61

*Added in 2015.

Few studies have systematically explored affordability and access to these newer drugs in SSA in reference to health financing metrics for specific countries.^17-19^ Our aim is to evaluate cancer medicine access in SSA as a function of both inclusion on the NEML and affordability by both governments and individual patients, who often pay for these medications out-of-pocket (OOP). We conducted these analyses using publicly available data from Kenya, Rwanda, and Uganda, focusing on the 10 most common cancers. We selected these 3 countries in East Africa because each had a different mechanism for financing cancer medicines. Medications on the NEML are covered in full by the government in Uganda, only for patients who purchase government health insurance in Kenya, and through a partnership with a non-profit organization with no cost-sharing by the government in Rwanda.

## Methods

### Population, Health Care, and Financing Metrics

Population and health care metrics including per capita gross national income (GNI), healthcare spending expenditure, and respective proportions of internal and external funding for healthcare were obtained from the World Bank database and World Health Organization databases.^20,21^ Data on cancer incidence and mortality was obtained from GLOBOCAN 2020.^1^

### NEMLs Alignment with the WHO EML

The 2019 WHO EML was used as a reference for comparison of the most recent WHO registered lists of national essential medicines for Kenya, Rwanda, and Uganda.^22-24^ Data were extracted from the NEMLs by 2 authors, with drugs limited to cytotoxic/antineoplastic agents, cancer supportive treatments, hormone and anti-hormone agents, targeted therapies, and immunotherapies. Extraction was limited to the drug name and used to provide a descriptive analysis of the proportion of WHO EML drugs listed on NEML for treatment of the 10 most common cancers in each country. If the NEML was published prior to 2019, a sensitivity analysis was done comparing alignment with the WHO EML published closest to the publication year of the NEML.

### Affordability of Cancer Medicines

The annual cost of a treatment regimen per patient for the 10 most common cancers in each country was estimated using the 2019 WHO EML supplementary cancer treatment guidelines,^13^ the median buyer price for a unit of medication, available through “Management Sciences for Health (MSH) International Drug Price Indicator Guide—2015,”^25^ and methodology and assumptions described in our prior study.^26^ When the price of a drug was not available in the 2015 MSH, the lowest available price (US pricing) was used as listed on UptoDate .^27^ Given large variations in cancer drug prices by world/income region,^28^ we performed sensitivity analysis compared median price listed on the MSH to listed prices on UptoDate and used descriptive analysis to summarize differences in price and assumptions made in our estimates. For metastatic disease, the duration of treatment for a particular regimen was determined based on median progression-free survival (PFS) from the respective clinical trials.

Most countries in SSA do not have universal health coverage (UHC) for cancer medicines,^29^ and patients in Kenya,^30-33^ Uganda,^33,34^ and Rwanda,^35,36^ may have to pay for medications OOP in the private sector when they are not available in the public sector. Thus, we assessed both the affordability of cancer treatment if purchased through UHC funded by the respective governments and OOP costs to individual patients if privately purchased. Using the WHO threshold for program cost-effectiveness, drug regimens where the annual cost of treatment per patient was higher than 3× the GNI per capita, were considered unaffordable. Since we regard to access to cancer care as a human right, for annual treatment costs exceeding 3× GNI per capita, we estimated price reductions required to ensure affordability (ie. ratio ≤ 3).^37^ Affordability of cancer medicines to patients paying OOP was assessed by using the WHO and Health Access Initiative (HAI) methodology, where a 30-day treatment course that cost more than the daily wage of the lowest unskilled government worker, was considered unaffordable.^38^ Since information about the wage of the lowest unskilled government worker was not available, a conservative estimate of daily wage was estimated using publicly available annual minimum wages for the respective countries.^39-41^

## Results

### Population, Health Care, and Financing Metrics

Kenya’s population was 52.6 million in 2019 compared to 44.2 million in Uganda and 12.6 million in Rwanda.^20^ In 2019, Kenya had a GNI per capita of $1750 compared to $780 in Uganda and $830 in Rwanda.^20^ Annual minimum wage was $1070.7 in Kenya,^39^ compared to $436.8 in Uganda,^40^ and $639.84 in Rwanda.^41^

### Alignment of NEML with the WHO EML

The Kenya 2019 NEML had the highest alignment with the WHO EML and included 93.4% of the 61 cancer medications listed in the 2019 WHO EML. The proportion of the 2019 WHO cancer medicines available on the 2016 Uganda NEML and the Rwanda 2015 NEML were 70.5% and 41.0%, respectively. However, there was 85.4% and 47.9% alignment when compared to the 2015 WHO EML, respectively. The Kenya NEML did not include all medicines on the 2019 WHO EML recommended for the treatment of one of its 10 most common cancers (leukemia), compared to 4 for Uganda (leukemia, lymphoma, breast cancer, and prostate cancer), and 5 in Rwanda (leukemia, lymphoma, breast, prostate, and lung cancer). (Table 1).

### Affordability of Cancer Medicines

**Figure 1.**
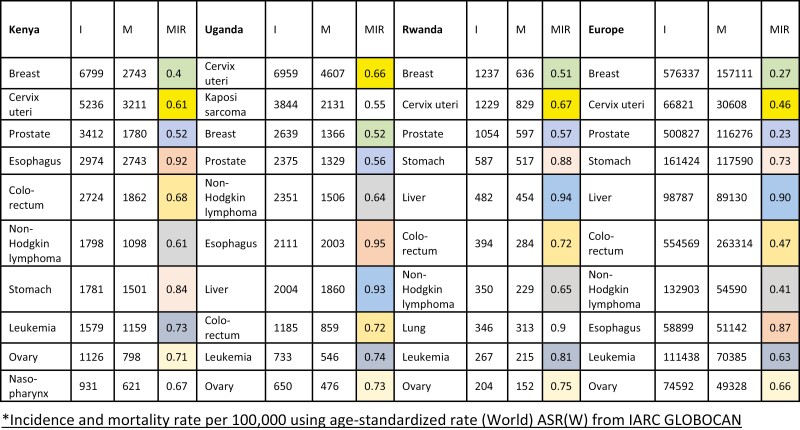
Ten most common cancers by incidence (I), including mortality (M) and mortality to incidence ratio (MIR) in Kenya, Uganda, and Rwanda, compared to trends for the same cancers in Europe.^1^*Incidence and mortality rate per 100 000 using age-standardized rate (World) ASR(W) from IARC GLOBOCAN*

Cervical, breast, prostate, colorectal, ovarian cancer, non-Hodgkin’s lymphoma, and leukemia were 7 of the top 10 cancers in all 3 countries that had recommended regimens on the WHO EML. (Fig. 1). There are no WHO EML indications for liver cancer, stomach cancer, and esophagus cancer. Mortality-to-incidence ratios (MIR) for these cancers were similar in all 3 countries. For comparison, MIRs for all these cancers except for liver, stomach, and esophagus cancer were lower in Europe compared to Kenya, Uganda, and Rwanda (Fig. 1).

Generic cytotoxic medications were available to treat 9 of the 13 most diagnosed cancers (7 in common between Kenya, Uganda, and Rwanda, and 6 others that varied across countries), and were all assessed as being affordable to governments if procured through a funded UHC program (Tables 3 and 4). All currently recommended treatments for prostate cancer, including leuprolide, bicalutamide, and abiraterone were not affordable through government procurement mechanisms. In addition, treatment regimens that included bendamustine, trastuzumab, rituximab, imatinib, dasatinib, and EGFR tyrosine kinase inhibitors (TKIs), were not affordable through government purchasing in all 3 countries, while capecitabine-based regimens were not affordable through government purchasing in Uganda and Rwanda only (Tables 2 and 3).

**Table 2.** Estimated chemotherapy costs, affordability, and availability of all cancer medications on the NEMLs.

Cancer type and stage	Regimen	Cost per patient per year (US$)	Affordability through government procurement		
			Kenya	Uganda	Rwanda
Breast; early-stage; TNBC	AC × 4 → T × 12	293	0.17	0.38	0.35
Breast; early-stage; ER+ and/or PR+, HER2-	AC × 4 → T × 12 with tamoxifen	338	0.19	0.43	0.41
Breast; metastatic; ER+ and/or PR+, HER2-	Tamoxifen	44	0.03	0.06	0.05
Breast; metastatic; ER+ and/or PR+, HER2-	Anastrozole	190	0.11	0.24	0.23
Breast; metastatic; TNBC	Paclitaxel × 8, capecitabine × 8	1766	1.01	2.26	2.13
Cervix, early-stage	Cisplatin weekly × 6 with RT	87	0.05	0.11	0.1
Colon cancer, stage II high-risk or Stage III	FOLFOX6 × 12	2132	1.22	2.73	2.57
Colon cancer, metastatic	FOLFOX × 8, then FOLFIRI × 8	1749	1	2.24	2.11
Non-Hodgkin lymphoma, DLBCL or FL	CHOP × 6	195	0.11	0.25	0.23
CLL/SLL, advanced symptomatic disease	CVP × 6, rituximab not available	130	0.07	0.17	0.16
Acute myeloid leukemia	7 + 3, HIDAC consolidation	734	0.42	0.94	0.88
CML, chronic phase	Nilotinib	1303	0.74	1.67	1.57
CML, accelerated phase or imatinib resistance	Nilotinib	1737	0.99	2.23	2.09
Ovarian cancer, early-stage, initial treatment	Carboplatin/paclitaxel × 6	745	0.43	0.96	0.9
Ovarian cancer, platinum-sensitive relapse	Carboplatin/paclitaxel × 6	745	0.43	0.96	0.9
Kaposi sarcoma	Paclitaxel × 6	199	0.11	0.26	0.24
Kaposi sarcoma, paclitaxel not available/toxicity	Vincristine/bleomycin × 6	178	0.1	0.23	0.21
Kaposi sarcoma, paclitaxel not available/toxicity	ABV × 6	211	0.12	0.27	0.25
Non–small cell lung cancer, stage I-II	Carboplatin/paclitaxel × 4	497	0.28	0.64	0.6
Non–small cell lung cancer, stage III	Carboplatin/paclitaxel weekly with RT × 4, then full dose × 2	505	0.29	0.65	0.61
Non–small cell lung cancer, stage V, no/unknown EGFR	Carboplatin/paclitaxel × 6	745	0.43	0.96	0.9
Nasopharyngeal cancer, early stage	Cisplatin weekly × 6 with RT	86	0.05	0.11	0.1
Nasopharyngeal cancer, metastatic	Carboplatin/paclitaxel × 7	1407	0.8	1.8	1.7
Rectal cancer, early stage	5-FU weekly with RT, then FOLFOX6 × 8	2361	1.35	3.03	2.84
Breast; early-stage; ER+ and/or PR+, HER2-	AC × 4 → T × 12, goserelin/anastrozole × 6 months	3562	2.04	4.57	4.29
Colon cancer, stage II high-risk or Stage III	CAPOX × 12	2928	1.67	3.75	3.53
Colon cancer, metastatic	CAPOX × 6, then FOLFIRI × 8	2736	1.56	3.51	3.3
Rectal cancer, early stage	Capecitabine weekly with RT, then FOLFOX6 × 8	3604	2.06	4.62	4.34
Prostate cancer, metastatic, castrate-sensitive	Bicalutamide × 30d, leuprolide, docetaxel × 6	7089	4.05	9.09	8.54
Non-Hodgkin lymphoma, DLBCL or FL	R-CHOP × 6	7232	4.13	9.27	8.71
Non-Hodgkin lymphoma, FL, advanced or symptomatic disease, grade 1-3a	R-CVP × 6	7168	4.1	9.19	8.64
CLL/SLL	FCR × 6	10 097	5.77	12.95	12.17
CLL/SLL, advanced symptomatic disease	R-CVP × 6	7168	4.1	9.19	8.64
Chronic myeloid leukemia (CML), chronic phase	Imatinib	9202	5.26	11.8	11.09
Breast; early-stage; ER-PR-HER2+	TCH × 6, then trastuzumab × 13	75 804	43.32	97.18	91.33
Breast; early-stage; ER+ and/or PR+, HER2+	ACx4 → TH × 6, then trastuzumab × 13 + tamoxifen	75 867	43.35	97.26	91.41
Breast; early-stage; ER+ and/or PR+, HER2+	TCH × 6, then trastuzumab × 13 + tamoxifen	75 849	43.34	97.24	91.38
Breast; metastatic; ER+ and/or PR+, HER2+	Docetaxel × 6, trastuzumab × 18	75 466	43.12	96.75	90.92
Breast; metastatic; ER-PR-HER2+	Docetaxel × 6, trastuzumab × 18, anastrozole	75 276	43.01	96.51	90.69
CLL/SLL	BR × 4	52 189	29.82	66.91	62.88
CML, accelerated phase or imatinib resistance	Dasatinib	221 347	126.48	283.78	266.68
Non-Hodgkin lymphoma, DLBCL or FL	BR × 6	78 283	44.73	100.36	94.32
Non–small cell lung cancer, stage V, EGFR activating mutation	Erlotinib	16 679	9.53	21.38	20.09
Non–small cell lung cancer, stage V, EGFR activating mutation	Gefinib	112 162	64.09	143.8	135.13
Prostate cancer, metastatic, castrate-resistant	Bicalutamide × 30d, leuprolide, abiraterone/prednisone	12 639	7.22	16.2	15.23

Cost reduction to reach 3:1 Cost/GNI ratio (Green—affordable; yellow—not affordable requiring up to 50% reduction; red—not affordable requiring >50% reduction).

Abbreviations: 5-FU, 5-fluorouracil; 7 + 3, cytarabine x 7 days, daunorubicin x 3 days; ABV, doxorubicin, bleomycin, vincristine; AC, doxorubicin cyclophosphamide; Adriamycin, doxorubicin; Ara-C, cytarabine; HiDAC, high-dose cytarabine consolidation; BR, bendamustine, rituximab; CAPOX, capecitabine, oxaliplatin; CLL, chronic lymphocytic leukemia; SLL: small lymphocytic lymphoma; CML, chronic myelogenous leukemia; CVP, cyclophosphamide, vincristine, prednisolone; DLBCL, diffuse large B-cell lymphoma; ER, estrogen receptor; FCR, fludarabine, cyclophosphamide, rituximab; FL, follicular lymphoma; R-CHOP, rituximab, cyclophosphamide, hydroxydaunorubicin hydrochloride (doxorubicin hydrochloride), oncovin (vincristine), prednisone; FOLFIRI, 5-fluorouracil, leucovorin, irinotecan; FOLFOX, 5-fluorouracil, leucovorin, oxaliplatin; Oncovin, vincristine (delete, redundant); PR, progesterone receptor; R, rituximab; RT, radiation therapy; T, taxol, either paclitaxel or docetaxel; TCH, docetaxel, carboplatin, transtuzumab (herceptin); TH, paclitaxel, herceptin; TNBC, triple-negative breast cancer.

**Table 3.** Estimated chemotherapy affordability and cost reduction to reach 3:1 Cost/GNI ratio

		Cost per patient per year (US$)	Cost per GNI for each country			Cost reduction required for affordability		
Cancer type and stage	Regimen		Kenya	Uganda	Rwanda	Kenya (%)	Uganda (%)	Rwanda
Breast; early-stage; TNBC	AC × 4 → T × 12	294	0.17	0.38	0.35	None	None	None
Breast; early-stage; ER+ and/or PR+, HER2-	AC × 4 → T × 12 with tamoxifen	338	0.19	0.43	0.41	None	None	None
Breast; metastatic; ER+ and/or PR+, HER2-	Tamoxifen	45	0.03	0.06	0.05	None	None	None
Breast; metastatic; ER+ and/or PR+, HER2-	Anastrozole	190	0.11	0.24	0.23	None	None	None
Breast; metastatic; TNBC	Paclitaxel × 8, capecitabine × 8	1766	1.01	2.26	2.13	None	None	None
Cervix, early-stage	Cisplatin weekly × 6 with RT	86	0.05	0.11	0.1	None	None	None
Colon cancer, stage II high-risk or stage III	FOLFOX6 × 12	2132	1.22	2.73	2.57	None	None	None
Colon cancer, metastatic	FOLFOX × 8, then FOLFIRI × 8	1749	1	2.24	2.11	None	None	None
Non-Hodgkin lymphoma, DLBCL or FL	CHOP × 6	195	0.11	0.25	0.23	None	None	None
CLL/SLL, advanced symptomatic disease	CVP × 6, rituximab not available	130	0.07	0.17	0.16	None	None	None
Acute myeloid leukemia	7 + 3, HIDAC consolidation	734	0.42	0.94	0.88	None	None	None
CML, chronic phase	Nilotinib	1303	0.74	1.67	1.57	None	None	None
CML, accelerated phase or imatinib resistance	Nilotinib	1737	0.99	2.23	2.09	None	None	None
Ovarian cancer, early-stage, initial treatment	Carboplatin/paclitaxel × 6	745	0.43	0.96	0.9	None	None	None
Ovarian cancer, platinum-sensitive relapse	Carboplatin/paclitaxel × 6	745	0.43	0.96	0.9	None	None	None
Kaposi sarcoma	Paclitaxel × 6	199	0.11	0.26	0.24	None	None	None
Kaposi sarcoma, paclitaxel not available/toxicity	Vincristine/bleomycin × 6	178	0.1	0.23	0.21	None	None	None
Kaposi sarcoma, paclitaxel not available/toxicity	ABV × 6	211	0.12	0.27	0.25	None	None	None
Non-small cell lung cancer, stages I-II	Carboplatin/paclitaxel × 4	497	0.28	0.64	0.6	None	None	None
Non-small cell lung cancer, stage III	Carboplatin/paclitaxel weekly with RT × 4, then full dose × 2	505	0.29	0.65	0.61	None	None	None
Non-small cell lung cancer, stage V, no/unknown EGFR	Carboplatin/paclitaxel × 6	745	0.43	0.96	0.9	None	None	None
Nasopharyngeal cancer, early stage	cisplatin weekly × 6 with RT	86	0.05	0.11	0.1	None	None	None
Nasopharyngeal cancer, metastatic	carboplatin/paclitaxel × 7	1407	0.8	1.8	1.7	None	None	None
Breast; early-stage; ER+ and/or PR+, HER2-	AC × 4 → T × 12, goserelin/anastrozole × 6 months	3562	2.04	4.57	4.29	None	34	30
Breast; early-stage; ER-PR-HER2+	TCH × 6, then trastuzumab × 13	75 804	43.32	97.18	91.33	93	97	97
Breast; early-stage; ER+ and/or PR+, HER2+	ACx4 → TH × 6, then trastuzumab × 13 + tamoxifen	75 867	43.35	97.26	91.41	93	97	97
Breast; early-stage; ER+ and/or PR+, HER2+	TCH × 6, then trastuzumab × 13 + tamoxifen	75 849	43.34	97.24	91.38	97	97	97
Breast; metastatic; ER+ and/or PR+, HER2+	Docetaxel × 6, trastuzumab × 18	75 466	43.12	96.75	90.92	93	97	97
Breast; metastatic; ER-PR-HER2+	Docetaxel × 6, trastuzumab × 18, anastrozole	75276	43.01	96.51	90.69	93	97	97
Prostate cancer, metastatic, castrate-sensitive	Bicalutamide × 30 d, leuprolide, docetaxel × 6	7089	4.05	9.09	8.54	26	67	65
Prostate cancer, metastatic, castrate-resistant	Bicalutamide × 30 d, leuprolide, abiraterone/prednisone	12 639	7.22	16.2	15.23	58	81	80
Colon cancer, stage II high-risk or stage III	CAPOX × 12	2928	1.67	3.75	3.53	None	20	15
Colon cancer, metastatic	CAPOX × 6, then FOLFIRI × 8	2738	1.56	3.51	3.3	None	15	9
Rectal cancer, early stage	5-FU weekly with RT, then FOLFOX6 × 8	2361	1.35	3.03	2.84	None	1	None
Rectal cancer, early stage	Capecitabine weekly with RT, then FOLFO6 × 8	3604	2.06	4.62	4.34	None	35	31
Non-Hodgkin lymphoma, DLBCL or FL	R-CHOP × 6	7232	4.13	9.27	8.71	27	68	68
Non-Hodgkin lymphoma, DLBCL or FL	BR × 6	78 283	44.73	100.36	94.32	93	97	97
Non-Hodgkin lymphoma, FL, advanced or symptomatic disease, Grade 1-3a	R-CVP × 6	7168	4.1	9.19	8.64	27	67	65
CLL/SLL	BR × 4	52 189	29.82	66.91	62.88	90	96	95
CLL/SLL	FCR × 6	10 097	5.77	12.95	12.17	48	77	75
CLL/SLL, advanced symptomatic disease	R-CVP × 6	7168	4.1	9.19	8.64	27	67	65
Chronic myeloid leukemia (CLL), chronic phase	Imatinib	9202	5.26	11.8	11.09	43	75	73
CML, accelerated phase or imatinib resistance	Dasatinib	221 347	126.48	283.78	266.68	98	99	99
Non-small cell lung cancer, stage V, EGFR activating mutation	Erlotinib	16 679	9.53	21.38	20.09	69	86	85
Non-small cell lung cancer, stage V, EGFR activating mutation	Gefinib	112 162	64.09	143.8	135.13	95	98	98

**Table 4.** Number of days wages needed for individuals paying OOP to buy a 30-day regimen of cancer treatment (Key: Yellow—≥30 days; salmon pink—>30-180 days; red—>180-365; dark gray—>365 days)

		Cost of 30 days of treatment	Number of days wages needed to pay for specified therapies		
Cancer type and stage	Regimen		Kenya	Uganda	Rwanda
Breast; early-stage; TNBC	AC × 4 → T × 12	24	8	20	14
Breast; early-stage; ER+ and/or PR+, HER2-	AC × 4 → T × 12 with tamoxifen	28	10	24	16
Breast; metastatic; ER+ and/or PR+, HER2-	Tamoxifen	4	1	3	2
Breast; metastatic; ER+ and/or PR+, HER2-	Anastrozole	16	5	13	9
Cervix, early-stage	Cisplatin weekly × 6 with RT	7	2	6	4
Non-Hodgkin lymphoma, DLBCL or FL	CHOP × 6	16	6	14	9
CLL/SLL, advanced symptomatic disease	CVP × 6, rituximab not available	11	4	9	6
Kaposi sarcoma	Paclitaxel × 6	17	6	14	9
Kaposi sarcoma, paclitaxel not available/toxicity	Vincristine/bleomycin × 6	15	5	12	8
Kaposi sarcoma, paclitaxel not available/toxicity	ABV × 6	18	6	15	10
Nasopharyngeal cancer, early stage	Cisplatin weekly × 6 with RT	7	2	6	4
Non-small cell lung cancer, stage I-II	Carboplatin/paclitaxel × 4	41	14	35	24
Non-small cell lung cancer, stage III	Carboplatin/paclitaxel weekly with RT × 4, then full dose × 2	42	14	35	24
Acute myeloid leukemia	7 + 3, HIDAC consolidation	61	21	51	35
Ovarian cancer, early-stage, initial treatment	Carboplatin/paclitaxel × 6	62	21	52	35
Ovarian cancer, platinum-sensitive relapse	Carboplatin/paclitaxel × 6	62	21	52	35
Non-small cell lung cancer, stage V, no/unknown EGFR	Carboplatin/paclitaxel × 6	62	21	52	35
Breast; metastatic; TNBC	Paclitaxel × 8, capecitabine × 8	147	50	123	84
Colon cancer, stage II high-risk or stage III	FOLFOX6 × 12	178	61	148	101
Colon cancer, metastatic	FsOLFOX × 8, then FOLFIRI × 8	146	50	122	83
CML, chronic phase	Nilotinib	109	37	91	62
CML, accelerated phase or imatinib resistance	Nilotinib	145	49	121	83
Nasopharyngeal cancer, metastatic	Carboplatin/paclitaxel × 7	117	40	98	67
Rectal cancer, early stage	5-FU weekly with RT, then FOLFOX6 × 8	197	67	164	112
Breast; early-stage; ER+ and/or PR+, HER2-	AC × 4 → T × 12, goserelin/anastrozole × 6 months	297	101	248	169
Colon cancer, stage II high-risk or stage III	CAPOX × 12	244	83	204	139
Colon cancer, metastatic	CAPOX × 6, then FOLFIRI × 8	228	78	191	130
Rectal cancer, early stage	Capecitabine weekly with RT, then FOLFO6 × 8	300	102	251	171
Prostate cancer, metastatic, castrate-sensitive	Bicalutamide × 30d, Leuprolide, docetaxel × 6	591	201	494	337
Non-Hodgkin lymphoma, DLBCL or FL	R-CHOP × 6	603	205	504	344
Non-Hodgkin lymphoma, FL, advanced or symptomatic disease, Grade 1-3a	R-CVP × 6	597	204	499	341
CLL/SLL, advanced symptomatic disease	R-CVP × 6	597	204	499	341
Prostate cancer, metastatic, castrate-resistant	Bicalutamide × 30d, leuprolide, abiraterone/prednisone	1053	359	880	601
CLL/SLL	FCR × 6	841	287	703	480
Chronic myeloid leukemia (CLL), chronic phase	Imatinib	767	261	641	437
Breast; early-stage; ER-PR-HER2+	TCH × 6, then trastuzumab × 13	6317	2153	5279	3604
Breast; early-stage; ER+ and/or PR+, HER2+	ACx4 → TH × 6, then trastuzumab × 13 + tamoxifen	6322	2155	5283	3607
Breast; early-stage; ER+ and/or PR+, HER2+	TCH × 6, then trastuzumab × 13 + tamoxifen	6320	2155	5282	3606
Breast; metastatic; ER+ and/or PR+, HER2+	Docetaxel × 6, trastuzumab × 18	6288	2144	5255	3587
Breast; metastatic; ER-PR-HER2+	Docetaxel × 6, trastuzumab × 18, anastrozole	6273	2138	5242	3578
Burkitt lymphoma/leukemia	R-Hyper-CVAD × 6, IT methotrexate × 4	1608	548	1344	917
CLL/SLL	BR × 4	4349	1483	3634	2481
Acute lymphoblastic leukemia, adult patients	R-Hyper-CVAD × 6, IT methotrexate × 4	1608	548	1344	917
CML, accelerated phase or imatinib resistance	Dasatinib	18 446	6288	15414	10522
Non-Hodgkin lymphoma, DLBCL or FL	BR × 6	6524	2224	5451	3721
Non-small cell lung cancer, stage V, EGFR activating mutation	Erlotinib	1390	474	1161	793
Non-small cell lung cancer, stage V, EGFR activating mutation	Gefinib	9347	3186	7810	5332

All WHO EML recommended medications used for the treatment of the top 10 cancers in Kenya and Uganda that were not listed as part of these NEMLs were also unaffordable to national governments without significant price reductions. The exception was nilotinib for CML, which was assessed as affordable through government procurement in both Uganda and Rwanda. Treatment regimens used for metastatic breast cancer, leukemia, and non-small cell lung cancer and that were not part of Rwanda’s NEML were below the affordability threshold (Tables 2 and 3) None of the treatment regimens for all the 10 most common cancer diagnoses for which there was a WHO EML indication were affordable to patients if paying out of pocket. (Table 4)

### Sensitivity Analysis

For the 15 medications examined, MSH and UptoDate price ranges overlapped in 6 (40%)—doxorubicin, paclitaxel, docetaxel, carboplatin, imatinib, and zoledronic acid. For those without overlap, UptoDate prices were 3.7-6.6× higher for rituximab, 6.4-6.9× higher for erlotinib, 7× higher for anastrozole, 14× higher for capecitabine, 16.5× higher for tamoxifen, 12.2-15.5× higher for cyclophosphamide, at least 35.7× higher for bicalutamide, and 150× higher for nilotinib. For medicines on both MSH and UptoDate, we used the lower MSH pricing. However, affordability estimates for medications not in MSH, including trastuzumab, bendamustine, goserelin, leuprolide, abiraterone, dasatinib, and gefitinib, may be higher compared to actual costs assessed in-country.

## Discussion

In this study, we used WHO tools for cost assessments and applied it to cancer care to evaluate cancer medicines access and affordability of WHO essential medicines for cancer treatment in SSA, drawing on data from Kenya, Rwanda, and Uganda and focusing on the 10 most common cancers in each country. Overall, NEML alignment varied across the different East African countries, even after consideration of the different years of NEML publication. Furthermore, cancer medicines were only affordable through government-financed procurement, such as through a UHC program, and no medicines were affordable through OOP individual purchases from patients. Newer targeted therapies were not affordable using current cost estimates and will require substantial reductions to make them affordable even through government purchasing.

Generic cytotoxic chemotherapies and hormone therapies included in the WHO EML were affordable to governments of the 3 countries, except for some capecitabine-based regimens in Uganda and Rwanda, which were not affordable for government purchasing. Newer targeted medicines and immunotherapies, except for nilotinib, were not affordable to the 3 country governments without significant price reductions of up to 99% for some drugs. Despite the high prices, all the newly added therapies are listed on the Kenya NEML except for dasatinib. Trastuzumab, rituximab, and imatinib are also part of the Rwanda NEML, while rituximab, leuprolide, bicalutamide, and capecitabine were listed on the Uganda NEML. None of the cancer treatment regimens were affordable to patients paying OOP, however ESMO data,^42^ suggests that most patients in SSA pay OOP for cancer medicines, which would thus force many patients in the 3 countries to forgo basic necessities or result in foregoing treatment, resulting in financial toxicity and potentially avoidable deaths.

This study has several limitations. We used publicly available data, which may not represent the actual availability and pricing of medications in the respective countries. However, the MSH median price index considers multiple countries and sources of price data and has been used in prior studies.^18,26^ In addition, when we compared prices available on UptoDate and prices listed in MSH, there was overlap in ranges of generic chemotherapies and less so targeted therapies, suggesting that our results for regimens that included abiraterone, trastuzumab, leuprolide, goserelin, dasatinib, gefitinib should be interpreted with caution. Ultimately, given that actual medication prices can vary widely,^19^ including within countries,^19,43^ with countries in Africa paying higher prices for anti-cancer drugs,^28^ these studies will be strengthened by follow-up surveys of actual pricing and availability to inform cancer procurement policies for the specific countries. Furthermore, cancer outcomes depend on many factors, including multidisciplinary care coordination,^44^ access to surgery, and radiation therapy. Other treatment modalities and inpatient admissions may account for the bulk of cancer treatment costs.^31^ Therefore, we predict our analyses represent conservative estimates, and total cancer care costs will exceed those provided in this analysis. While we based our analysis on the 2019 WHO EML treatment indications focusing on the 10 most common cancers, we recognize that other guidelines could be used for the purpose, including the NCCN Harmonized Guidelines for Sub-Saharan Africa.^45^ Our analysis does not include medicines on the 2021 WHO EML which was recently published, and includes newer patented medicines,^46^ which are likely to not be affordable at current costs. Finally, mortality-to-incidence ratios for the most common cancers in all 3 countries were similar despite differences in cancer medicine access, and generally higher than mortality-to-incidence ratios for these cancers in Europe, likely reflecting differences in cancer early diagnosis and access to treatment. An analysis of the impact of cancer medication access on cancer outcomes is outside of the scope of our study.

Despite the above limitations, we believe that our results are robust and will be valuable to governments and other key stakeholders in SSA and other LMICs in selecting strategies to improve access and affordability of cancer medicines in their countries. This study is novel in providing a potential evaluation model for providing estimates of access and affordability of specific guideline-recommended cancer treatment regimens for the 10 most common cancers diagnosed in the specific countries. The analysis was applied to countries in Sub-Saharan Africa with different NEML cancer medicine financing mechanisms. This model can be extended readily to other countries in the region and to other low and middle-income countries (LMICs), as well as to cancers outside of the top 10 list.

It also provides further justification for including cancer treatment coverage as part of a UHC program, since our analysis supports that no cancer drugs or treatment regimens are affordable through OOP purchasing by individual patients. In fact, a recent study of 148 countries showed that one of the predictors of improved breast cancer survival was the increased coverage of essential health services for cancer care.^47^

In all 3 countries, there are ongoing efforts to make cancer treatment more affordable, including through partnerships with philanthropic foundations and working to expand access to chemotherapy as is being done in Rwanda.^35^ In Kenya, efforts are underway to negotiate drug prices with pharmaceutical companies and expand access to the National Health Insurance, which is purchased OOP and includes coverage for the equivalent of up to US $50 000 per lifetime for cancer treatment.^30^ Based on the 2017 European Society for Clinical Oncology (ESMO), patients in Kenya had to pay 100% of the retail cost for all the medications, except for capecitabine, irinotecan, and oxaliplatin, which were discounted 50-100%, and imatinib, which was free, suggesting that even generic drugs listed on the NEML may not be provided free of charge to patients by governments in the respective countries. However, a more recent study published in 2021 reported that all cancer medicines are free and available at the Uganda Cancer Institute.^34^ These efforts are necessary but remain insufficient to alleviate the financial toxicity associated with a cancer diagnosis in SSA.

Although inclusion in the NEML is the first step to increasing access, data suggests that it does not reflect the actual availability of the drugs at the point of care delivery. According to the 2017 ESMO survey, most medications from the 2015 WHO EML were “usually available” and only 6 “always available” in Kenya, with unreliable suppliers reported as the main barrier.^42^ In Uganda, about half of the medications on the 2015 WHO EML were reported as usually available and about half as always available, including imatinib, while irinotecan was only available half of the time, with barriers including unreliable suppliers, government capitation for capecitabine, irinotecan, oxaliplatin, trastuzumab; no commercial motive for imatinib and manufacturing problems for vinorelbine and cyclophosphamide tablet.^42^ A study done in Uganda in 2021 reported that 85.8% of all NEML medications were available as a result of a centralized national procurement system.^34^ No data is available about the availability of cancer medications in Rwanda. Studies suggest that stockouts in all 3 countries pose a challenge and result in patients paying higher OOP prices in the private sector if the free drugs are out of stock in the public sector.^19,32,33,36,48^

Our results highlight important gaps in the availability and affordability of cancer medicines that impair the ability of SSA countries to address the growing burden of cancer-associated morbidity and mortality. Several studies have used the WHO/HAI methodology to estimate the affordability of cancer medications to patients paying OOP in other LMICs. In India, pediatric lymphoma and leukemia were unaffordable OOP.^49^ In South Africa, analysis of individual drug prices and not regimens showed that the originator brand (OB) prices were affordable OOP only for paclitaxel 300 mg and fluorouracil 500 mg, while the lowest price generic (LPG) was affordable only for paclitaxel 300 mg, doxorubicin 10 mg, and oxaliplatin 100 mg in the private sector.^18^ A study in Kenya estimated that the entire cost of treatment for locally advanced breast and cervical cancer was unaffordable OOP and 6× higher in the private compared to the public sector.^19^

Cancer medicine affordability cannot be achieved solely through inclusion on the WHO EML^28^ or respective NEMLs. Potential regional, national, and international strategies to improve cancer medicine access include: (1) strengthening pricing policies across healthcare sectors, including price caps for cancer medicines and optimizing a healthcare system’s ability to review and adjust medication prices; (2) implementation of differential pricing based on healthcare systems’ purchasing power; (3) increasing transparency around cancer medicine prices^28,50^; (4) pooling resources between MOH and other key partners, including non-governmental organizations,^51,52^ the WHO, multilateral financing entities to subsidize healthcare budgets, and academic institutions^53^ for joint cancer drug price negotiation and pooled procurement^16,54^; (5) using voluntary and compulsory licensing and applying World Trade Organization Trade-Related Property Rights flexibilities to patented cancer medicines^54,55^; (6) promoting health services research and program implementation to improve system efficiencies related to cancer drug procurement,^26^ and rational use, including by partnering with philanthropic foundations^56^ and academic institutions^34,35^; and (7) removing incentives for prescribing cancer medicines of limited clinical value.^50,57,58^

Healthcare policies that make cancer medicines more accessible can improve population-level cancer outcomes when they are tailored to the country context, including cancer epidemiology and healthcare system financing, resources, and priorities.^50^ While significant work has been done to improve cancer medicine access in Kenya, Rwanda, and Uganda, our analysis points to additional specific steps forward that may be helpful in improving access and affordability of essential cancer medicines and ultimately clinical outcomes.

## Conclusion

Our study shows that cancer treatment regimens based on WHO EML indications that include novel cancer drugs are not affordable to LMIC governments in Sub-Saharan Africa, and almost all cancer treatment regimens are not affordable to individuals paying OOP. Thus, a universal health insurance scheme is essential to ensuring access to cancer care and treatment in countries of sub-Saharan Africa. As cancer emerges as an increasing global epidemic, more research about effective programs and interventions for improving access and reducing costs to cancer medicines is urgently needed.

## Data Availability

The data underlying this article will be shared on reasonable request to the corresponding author.
